# C2-addition patterns emerging from acetylene and nickel sulfide in simulated prebiotic hydrothermal conditions

**DOI:** 10.1038/s42004-023-01021-1

**Published:** 2023-10-12

**Authors:** Philippe Diederich, Alexander Ruf, Thomas Geisberger, Leopold Weidner, Christian Seitz, Wolfgang Eisenreich, Claudia Huber, Philippe Schmitt-Kopplin

**Affiliations:** 1 Helmholtz Munich, Research Unit Analytical BioGeoChemistry, Neuherberg, Germany; 2 Excellence Cluster ORIGINS, Boltzmannstraße 2, 85748 Garching, Germany; 3 LMU Munich, Faculty of Physics, Schellingstraße 4, 80799 Munich, Germany; 4 Technical University of Munich, TUM School of Natural Sciences, Department of Bioscience, Bavarian NMR Center (BNMRZ), Structural Membrane Biochemistry, Lichtenbergstr. 4, 85748 Garching, Germany; 5 Comprehensive Foodomics Platform, Chair of Analytical Food Chemistry, TUM School of Life Sciences, Technical University of Munich, Maximus-von-Imhof-Forum 2, 85354 Freising, Germany; 6 Center for Astrochemical Studies, Max Planck Institute for Extraterrestrial Physics, Gießebachstraße 1, 85748 Garching bei München, Germany

**Keywords:** Biogeochemistry, Origin of life, Mass spectrometry, Lipidomics

## Abstract

Chemical complexity is vital not only for the origin of life but also for biological evolution. The chemical evolution of a complex prebiotic mixture containing acetylene, carbon monoxide (CO), and nickel sulfide (NiS) has been analyzed with mass spectrometry as an untargeted approach to reaction monitoring. Here we show through isotopic 13C-labelling, multiple reaction products, encompassing diverse CHO and CHOS compounds within the complex reaction mixture. Molecules within the same chemical spaces displayed varying degrees of 13C-labelling, enabling more robust functional group characterization based on targeted investigations and differences in saturation levels among the described classes. A characteristic C2-addition pattern was detected in all compound classes in conjunction with a high diversity of thio acids, reminiscent of extant microbial C2-metabolism. The analysis involved a time-resolved molecular network, which unveiled the behavior of sulfur in the system. At the onset of the reaction, early formed compounds contain more sulfur atoms compared to later emerging compounds. These results give an essential insight into the still elusive role of sulfur dynamics in the origin of life. Moreover, our results provide temporally resolved evidence of the progressively increasing molecular complexity arising from a limited number of compounds.

## Abstract (web summary)

Various fatty acids and aldehydes can be synthesized from acetylene and carbon monoxide by nickel sulfide catalysts under volcanic hydrothermal conditions compatible with aqueous early earth conditions, however, their chemical complexity remains underexplored. Here, the authors use ^13^C-labelling together with untargeted ultrahigh-resolution mass spectrometry to categorize carboxylic acid functional groups, revealing C2-addition driven compound diversity as well as new sulfur containing classes.

## Introduction

In 1953, Reppe pioneered the synthesis of fatty acids from acetylene and carbon monoxide^1^. In conjunction with a nickel carbonyl catalyst, both gases yielded acrylic acid and longer unsaturated fatty acids. However, nickel carbonyl, a water-labile compound, is not the only catalyst capable of lowering the required energy to accelerate this reaction. Recently, this reaction was reinterpreted in an origin-of-life context as fatty acids play structural and energetic roles in living organisms. Experimental evidence that describes the formation of a variety of fatty acids of different lengths and degrees of saturation was achieved under volcanic hydrothermal conditions, utilizing a nickel sulfide catalyst compatible with aqueous early earth conditions^2^.

So far, acetylene has been underrepresented in origin-of-life research. Literature is scarce, but new evidence for its relevance emerges. Acetylene is formed from methane irradiated by UV light^3^. Multiple planetary bodies show an atmosphere partially made from acetylene gas, like the Saturn moon Titan^4,5^ and the Jovian planet Jupiter^6^. The atmosphere of Enceladus, another moon of Saturn, contains acetylene in addition to phosphorous^7^ and exhibits hydrothermal volcanic activity^8^. The existence of acetylene in the atmosphere of early Earth was also hypothesized^9^ and can be found nowadays in fumaroles of geothermal areas^10^. Spark discharge experiments of gaseous nitrogen and methane mixtures also lead to the formation of acetylene^11^. Extant microorganisms in anaerobic aqueous environments can use acetylene as an energy and carbon source, increasing the likelihood of its presence at early evolutionary stages. Specifically, *Pelobacter acetylenicus* can grow from acetylene^12^ alone and therefore demonstrates that acetylene has the potential to fuel a complete metabolism on its own. Acetylene is transformed into acetyl-coenzyme (Co)A, a thio ester, via the hydration of acetylene into acetaldehyde. Acetyl-CoA is then used to build metabolites with C2-units.

The role of sulfur in the origin of life is still an elusive and extensively researched topic. Undoubtedly sulfur is essential for extant life, as it is part of methionine and cysteine, acetyl-CoA, and multiple hydrogenases^13^. Popular hypotheses for the origin of life also include sulfur as a critical element in a more indirect way. Inspired by the “iron-sulfur world” theory of Wächtershäuser^14^, transition-metal sulfides were used as catalysts in origin-of-life experiments. This theory proposed a mineral surface metabolism, starting from simple inorganic precursors and evolving into complex bioorganic molecules. This hypothetical chemoautotrophic evolution proceeds *via* thio acids or thioesters in reductive autocatalytic cycles^15^. Incubation of carbon monoxide with methyl mercaptan over transition-metal sulfides leads to the well-coveted activated thioester^16^, a molecular part of acetyl-CoA. These seminal results sparked the discovery of other reactions, possible in a hydrothermal environment and yielding prebiotically-relevant compounds, including acetaldehyde^17^, Krebs cycle intermediates^18^, and the porphyrin building block pyrrole^19^. De Duve proposed a second hypothesis, heavily depending on sulfur compounds where thioesters contribute the energy for essential reactions^20^. Moreover, thiols were described as a possible prebiotic intermediate for peptide-bond formation^21^, whereas sulfur-containing heterocycles could act as catalysts for biologically-relevant reactions^22^.

A deeper comprehension of chemical complexity is vital not only for the origin of life but also for biological evolution. We combined ^13^C-labelling with untargeted ultrahigh-resolution mass spectrometry to tackle the challenging analysis of highly complex evolving abiotic systems. We describe an analytical approach based on known chemical reactions that allows us to categorize detected elemental compositions into individual compound classes via the degree of ^13^C-labelling. In this study, we expanded our knowledge of the diversity of compound classes formed from prebiotically relevant gases. We recognized C2-addition as a driving factor for compound diversity in a given compound class. In addition, we identified new functional classes, such as thio ethers and thio acids. Through labeling, we discovered that there are two pathways leading to thio acids, which are crucial molecular components of acetyl-CoA. Thioacids formed solely from acetylene also enabled comparison with Pelobacter acetyleneicus. Lastly, this approach succeeded in detecting a sulfur-specific trend that led to the reduction of sulfur atoms per molecule after the initial introduction of sulfur to the gases. This trend led to compounds with low numbers of sulfur, more aligned with biomolecules in extant organisms, shedding light on sulfur dynamics in abiotic systems.

## Results

### Compositional complexity increases over time

**Fig. 1:**
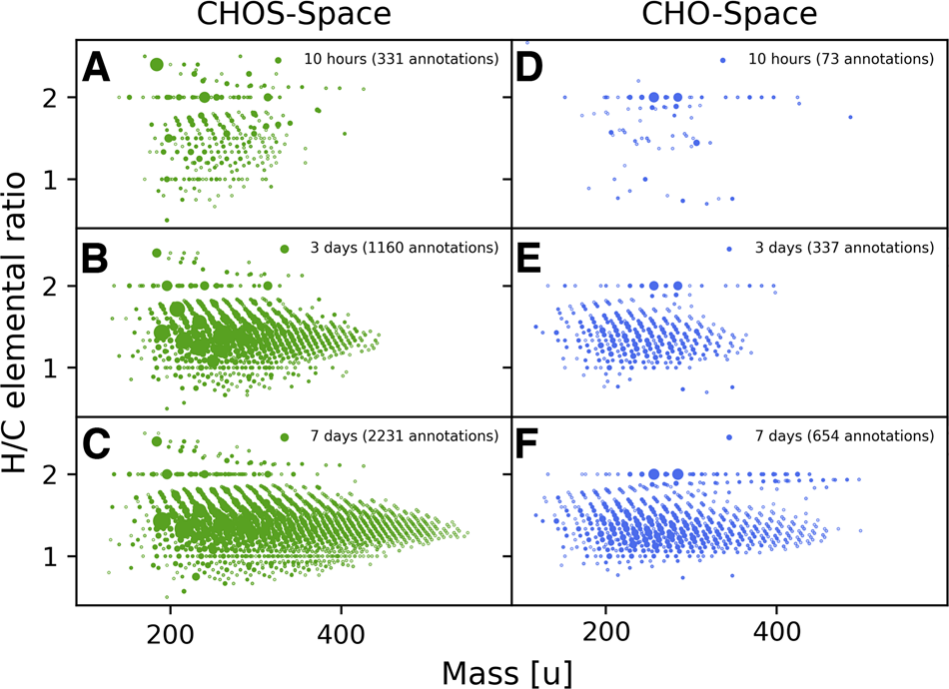
Overview of the CHO and CHOS space over time. Overview of two different chemical spaces within the reaction mixture (acetylene/CO/H_2_O/NiS). The annotated elemental compositions are plotted with the ratio of hydrogen atoms to carbon atoms (H/C) against the mass of the elemental composition. Different panels represent different time points for CHO (**D**–**F**, blue) and CHOS (**A**–**C**, green). Marker sizes represent relative detected intensity.

We incubated acetylene with carbon monoxide over water containing nickel sulfide for varying time periods at 105 °C and measured the evolving mixture at different time points. After 2 h, the first signals belonging to reaction products were analyzed by direct infusion Fourier transform ion cyclotron resonance mass spectrometry (FT-ICR-MS). Longer incubation times increased the chemical diversity and complexity of organic compounds, meaning a functional and compositional variety of molecules. Visualization of the increasing diversity of elemental CHO and CHOS compositions found in the one-pot reaction can be seen in Fig. 1. The mass-to-charge ratio was plotted against the hydrogen-to-carbon ratio. This separation allowed an overview of the chemical composition of the system resolved by the mass and the degree of saturation of the individual compounds. We separately focused on the chemical space of CHO and CHOS compounds. We observed an increase in detected signals over time for both chemical spaces, strongly suggesting a diversification of reaction products by a factor of ~9. The main group of the detected compounds is highly unsaturated, as the average H/C ratio is 1.4 for both chemical spaces after 7 days. This strong bias toward unsaturated compounds is in line with the utilized reactants (acetylene possessing a triple bond) and targeted GC-MS measurements of formed highly unsaturated carboxylic acids in the mixture^2^. Further, the mass of the compounds increased over time. The overall diversity increases as well, as the number of detected CHO and CHOS compounds increases from 328 annotated signals after 2 h to 2885 annotated signals present after 7 days.

The compositional space visualization of the ongoing reactions gives an overview of the system, but elemental compositions alone do not provide information about isomers and, more importantly, functional groups. The determination of such functional groups remains a determining factor if specific reactions need to be characterized.

### Resolving functional diversity by ^13^C-labelling

Comparing unlabeled setups to setups with ^13^C-labelled carbon monoxide allowed us to characterize functional groups and compound class diversity better. Categorizing the elemental annotations by ^13^C-labelling improved the amount of information, where ^13^C-carbons convey specific functional insights into the onset of an early evolving system. This additional dimension allowed us to resolve different compound classes with the same number of heteroatoms. The origin of the label (carbon monoxide in our experimental setup) provided insight into the functional groups present in elemental subspaces after chemical reactions. Targeted GC-MS analysis of this mixture revealed that carbon monoxide is mainly reacted into a carboxylic acid group^2^, in agreement with Reppe´s chemistry^1^.

**Fig. 2:**
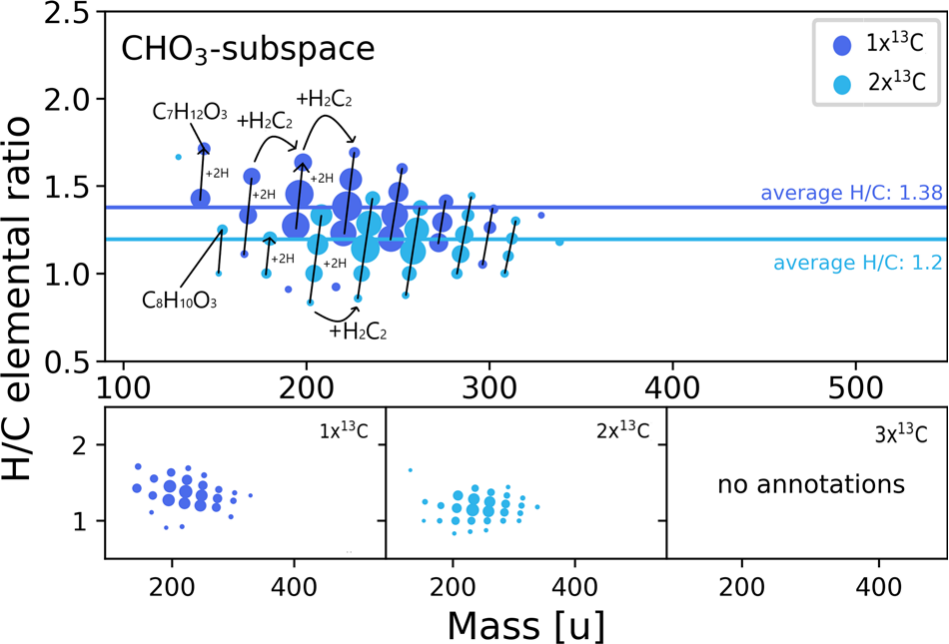
CHO_3_-subspace Elemental compositions in the C_x_H_y_O_3_-subspace colored by the degree of ^13^C-labelling. Differences in acetylene and hydrogen building blocks are highlighted by black arrows. Exemplary structures belonging to the identified compound class are shown for both degrees of ^13^C-labelling. Horizontal colored lines show the average H/C ratio for the different degrees of labeling. Marker sizes represent relative detected intensity.

In line with published data^2,18^, we were able to differentiate distinct populations of CHO and CHOS compounds in our model, as some compounds are labeled once, twice, or higher. C_x_H_y_O_3_-compounds form the first elemental composition with more heteroatoms than pure fatty acids (Fig. 2). The elemental compositions with a single ^13^C-label show a saturation level on average higher (1.38) than the saturation of the double-labeled compositions (1.20). This result strengthens the hypothesis that carbon monoxide is introduced as a carbonyl group. The carbonyl group reduces the H/C ratio compared to a hydroxy group or ether. Mono-labeled compounds, therefore, belong to carboxylic acids carrying an additional hydroxy or ether group. The addition of water to a double bond is likely responsible for the increase in oxygen in molecules without the need for carbon monoxide. We further observed that the number of oxygens in compounds with one ^13^C label positively correlates with the H/C ratio of the detected compounds, further strengthening the claim that the introduction of oxygen via water consumes a double bond (supplementary fig. 1). These results suggest a separation of the CHO_3_ space into keto acids and either hydroxy acids or carboxylic acids with an ether (cyclic unsaturated ethers like furans, for example). Similar carboxylated heterocycles with sulfur, namely thiophenes, were already described in the system^23^.

The recurring pattern of molecules reflecting C2-addition originating from acetylene is important to mention. Compounds of a specific subspace with the same amount of ^13^C label increase in mass only via the addition of acetylene or reduction of double bonds. This addition of C2-units is reminiscent of the C2-metabolism carried out by acetyl-CoA. This behavior of mass increase via acetylene can be seen for all identified compound classes.

**Fig. 3:**
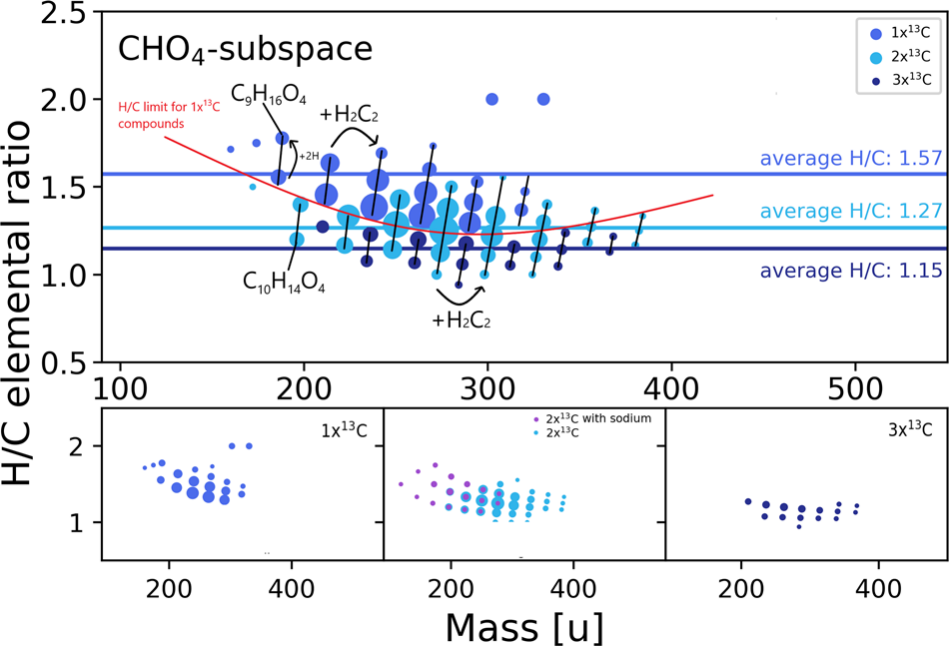
CHO_4_-subspace. Elemental compositions in the C_x_H_y_O_4_-subspace colored by the degree of ^13^C-labelling. Violet color shows sodium adducts. Red line marks the clear border in saturation between mono-and triple-labeled compounds. Differences in acetylene and hydrogen building blocks are highlighted by black arrows. Exemplary structures belonging to the identified compound class are shown for all degrees of ^13^C-labelling and adducts. Horizontal colored lines show the average H/C ratio for the different degrees of labeling. Marker sizes represent relative detected intensity.

Additional heteroatoms increase the number of potential isomers and functional groups. This labeling approach further allows distinguishing at least three different compound classes in the CHO_4_-subspace (Fig. 3). Mono-labeled compounds show the expected behavior of an increase in H/C-ratio compared to the CHO_3_-subspace (from 1.38 to 1.57). This is the result of the consumption of a double bond during the introduction of water into the molecule. The mono-labeled compounds belong to the group of dihydroxy-acids and were not yet described in the system. Double-labeled compounds present a challenge, as the labeling still allows the formation of dicarboxylic acids or hydroxy-keto acids. The information that carbon monoxide is converted into a carbonyl group is insufficient for categorization. Further categorization requires the inclusion of an additional element, namely sodium. Our data suggested that dicarboxylic acids are prone to form sodium adducts after negative electrospray ionization. A double-deprotonated species can carry a positively charged sodium ion while still being negatively charged. This observation allows for the separation of double-labeled compounds into two potential subgroups.

Compositions labeled twice and showing sodium adducts can be categorized as dicarboxylic acids. The presence of succinic acid in the system was already shown in a previous publication^18^. Notably, no annotations with a single label (tentatively one carboxylic group) and a sodium adduct are detected. The same applies to the triple-labeled compounds that will be discussed later. This fact strengthens the hypothesis that this behavior is specific to dicarboxylic acids in the CHO_4_ subspace. A further sign of the occurrence of dicarboxylic acids is the strongly increased average H/C ratio (1.39) of compounds falling into this category compared to the average of this class (1.27). The introduction of a further carboxylic group consumes a double bond if the underlying mechanism complies with the reaction hypothesis of Reppe. This consumption of a double bond would raise the saturation level of the compounds and explain the strong deviation from the remaining double-labeled annotations. The categorization is still ambiguous, as compounds with higher mass may lead to reduced formation of sodium adducts. Notably, all sodium adducts are detected in a lower mass range than non-sodium-adduct-forming compounds. Strong evidence, however, for the presence of a mix of dicarboxylic acids and hydroxy-keto acids in the double-labeled CHO_4_-subspace is the fact that a triple-labeled group is present. No sodium adducts are observed in this group, and the H/C ratio is, on average, lower (1.15). These results strengthen the annotation as diketo acids and make the presence of hydroxy-keto acids for double-labeled compounds more likely. CHO_5_ and CHO_6_ subspaces follow the same trends described for the previous subspaces.

**Fig. 4:**
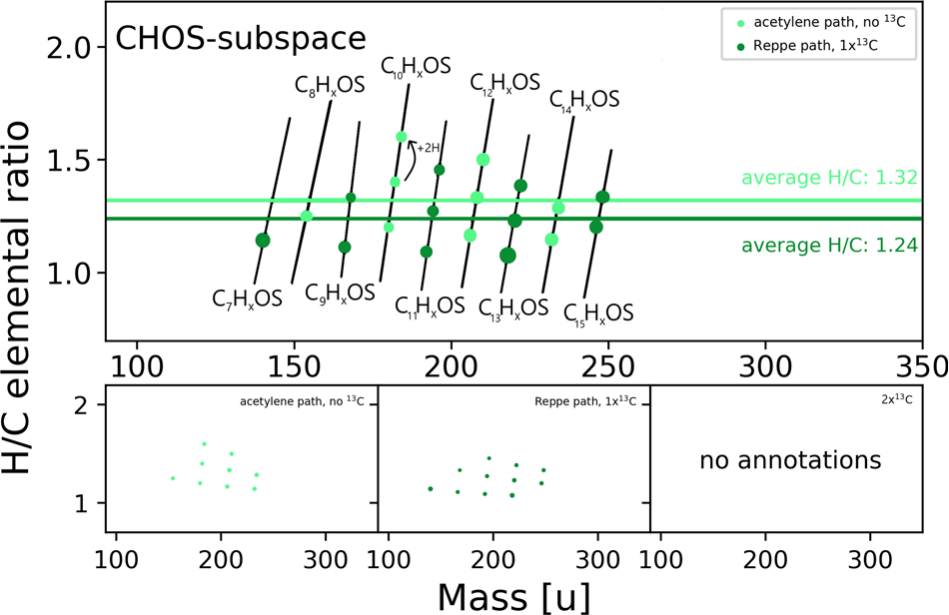
C_x_H_y_O_1_S_1_-subspace. Elemental compositions in the CxHyO1S1-subspace representing thio acids, colored by the degree of 13C-labelling. Black encircled dots could also be confirmed to consist solely of acetylene building blocks. Differences in hydrogen building blocks are highlighted by black arrows. Horizontal colored lines show the average H/C ratio for the different degrees of labeling. Marker sizes represent relative detected intensity.

Compounds containing sulfur show similar patterns. The CHO_1_S_1_ is the first elemental subspace in the category of CHOS compounds. Our results distinguished two groups (Fig. 4). Unlabeled compounds originate from acetylene alone, whereas mono-labeled compounds are derived from acetylene and a single carbon monoxide molecule. No double- or triple-labeled CHO_1_S_1_ compounds were detected. CHO_1_S_1_ compounds match the elemental composition of thio acids. The possibility of a modified Reppe chemistry with hydrogen sulfide instead of water leading to thio acids was already shown in earlier works of Reppe^1^. Having both labeled and unlabeled species of this compound class is surprising, as the pathway described by Reppe requires carbon monoxide. However, the probability that the unlabeled compounds belong to a different class of compounds is low, as hydroxy and thiol groups alone do not efficiently ionize in negative ionization mode. Those unlabeled species are also mostly fully labeled if acetylene is used with two labeled carbons instead of carbon monoxide (Fig. 4, black circled dots), excluding contamination. The formation of thio acetic acid could be detected in high amounts at early time points in the system with NMR (see Supplementary Fig. 2), further strengthening the presence of thio acids formed without carbon monoxide. If no more than two oxygen atoms are present in the molecule, the amount of labeled carbon does not exceed one ^13^C. This result suggests that sulfur cannot completely exchange oxygen in this system. Oxygen originating from carbon monoxide stays attached to its labeled carbon.

The increased number of hetero atoms in the CHOS subspaces progressively leads to ambiguity. Relevant information can still be gained from ^13^C labeling. The CHO_3_S subspace behaves similarly to the CHO_3_ rather than the CHO_4_ subspace. Only mono- and double-labeled compounds can be detected. In addition, no sodium adducts are detected. This result suggests that compounds carrying a carboxylic and a thiocarboxylic acid group simultaneously are absent from the system. CHO_4_S is similar in this regard. The diversity in sodium adducts for this subspace exceeds the diversity of the non-sodium-adduct-forming fraction of double-labeled compounds. However, the complete absence of triple-labeled compounds is very surprising in this subspace, likely because double-labeled CHO_4_S belong to thioethers. Two carboxylic acids are linked to each other via a sulfur ether. To further strengthen this claim, the detected elemental composition of C_6_H_10_O_4_S was identified and validated as thiobispropanoic acid via targeted GC-MS analysis by comparing retention time and the fragmentation pattern to a commercially available standard (Supplementary Fig. 3). Adding one sulfur to reach the CHO_4_S_2_ subspace shows only double-labeled sodium adducts as a coherent homologous series. The result suggests the presence of two carboxylic acids linked by a sulfur bridge, reminiscent of protein sulfur bridges allowing their tertiary structure (Supplementary Fig. 4).

Further interpretations of higher heteroatom combinations were deemed too ambiguous, but a table showing all annotations with the corresponding labels can be found as a supplementary Excel sheet (Supplementary Data 1). One source of ambiguity is the presence of “mixed” ^13^C annotations where the same elemental composition is present in different degrees of ^13^C labeling. Due to this, it is almost impossible to reliably assign functional groups to a detected signal. However, it is still interesting to investigate how the percentage of “mixed” annotations changes for different subspaces in such a complex mixture. A reaction scheme summarizing all proposed mechanisms and 13C-patterns can be found in S.I. (supplementary fig. 5).

### Isomer frequency has an unpredictable variance in analyzed subspaces

^13^C labeling of the sample allowed additional dimension for untargeted analysis, which is otherwise inaccessible in direct-infusion mass spectrometry. The labeling revealed that some of the identical elemental compositions existed as a mixture of ^13^C labeled species, as labeled experiments yielded signals compatible with multiple degrees of labeling. A representative comparison between labeled and unlabeled spectra can be found in S.I. (supplementary fig. 6). We investigated the percentage of mixed and pure annotations for different chemical subspaces and observed unexpected behaviors. With an increasing number of hetero atoms, we expected a steady increase in the fraction of mixed annotations compared to pure annotations. This, however, was not always the case. The CHO space showed an increase of mixed annotations from two (7.7%) to three (17.4%) oxygens per molecule. The CHO_4_-subspace, however, only shows 12.9%, and the CHO_5_ subspace 4.9% of mixed annotations. The CHOS space showed increased mixed annotations compared to the CHO space. However, no clear trend could be elucidated. CHO_1_S_1_ and CHO_2_S_3_ showed 0% mixed annotations, in contrast to CHO_3_S_2,_ with 39% mixed annotations. These results highlight the unpredictability of isomer populations in untargeted FT-ICR-MS.

Using ^13^C labeling allows a definite distinction between impurities and experimental sample components^24^. This result is difficult to achieve with FT-ICR-MS, as removing blank signals potentially removes specific results corresponding to compound isomers in solvents or other sources of contamination. In some instances, compounds like fatty acids can be in the solvent and the investigated system.

### Temporal evolution of the system

**Fig. 5:**
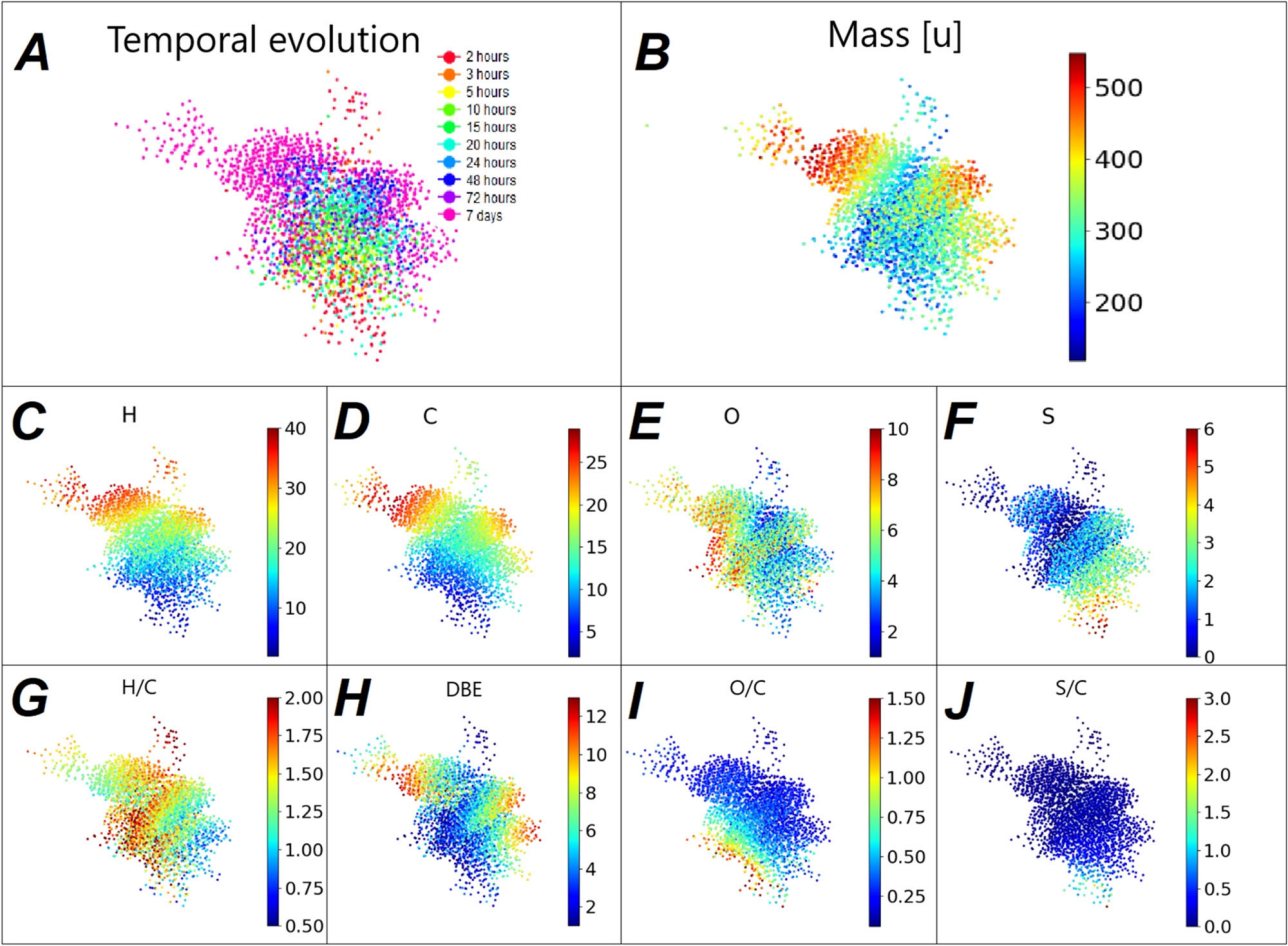
Molecular network of all annotated signal. **A** Time-resolved molecular network evolving from red (2 h) at the bottom to pink at the top (7 days). **B** Mass-resolved molecular network showing an increase in mass correlated with the increase in time. Molecular network colored by the number of assigned elements (**C**–**F**) or elemental ratios (**G**–**J**).

The system was visualized over time via a temporal molecular network (Fig. 5). In this network, individual nodes represent detected annotated signals. Overall, 6 elemental compositions were chosen as possible edges, all representing differences in molecular weight after specific reactions. Based on previous publications or available reactants, the 6 chosen mass differences (Table 1) represent possible reactions likely to happen in the system. Edges can represent the addition of acetylene^25^, the simultaneous addition of carbon monoxide and water to a double or triple bond to form a carboxylic acid group. The same reaction can be slightly altered with hydrogen sulfide instead of water resulting in a thio acid^1^. The addition of water^26^, hydrogen sulfide^27^, or hydrogen represents the loss of a double or triple bond, potentially through reduction. This limited number of possible edges allowed to interconnect 97.5% of all annotated signals.

**Table 1:** Mass differences used as edges in the molecular network to connect all annotated formulas.

Possible reaction	Elemental difference	Exact mass difference (u)
Water addition	+ H_2_O	18.01057
Hydrogen sulfide addition	+ H_2_S	33.98772
Dimerization/Oligomerization	+ C_2_H_2_	26.01565
Carbonylation (with H_2_O)	+ CH_2_O_2_	46.00548
Carbonylation (with H_2_S)	+ CH_2_OS	61.98264
Reduction	+ H_2_	2.01565

The overall temporal path taken by the system is visualized by a molecular network (Fig. 5A). Molecular networks and mass-difference analysis were developed for biological samples^28^ and used in recent untargeted investigations of astrochemical reactions^29^ allowing a comprehensive overview of the chemical system. The masses increase over time, and later detected molecules show the highest masses (>500 u) (Fig. 5B). The amount of hydrogen and carbon follow a nearly identical trend compared to the mass (Fig. 5C and D). The percentage of compounds containing high numbers of sulfur atoms per molecule (>2) sulfur) is highest at early time points (Fig. 5F). The percentage of CHO_x_S_3_ annotations starts at 29% of overall sulfur annotations after 2 h and is lowered to 17% after 7 days, even though the absolute amount of CHO_x_S_3_ annotations is increasing (from 60 annotations after 2 h to 388 after 7 days). CHO_X_S_4_ compounds start at 39 annotations after 2 h and go down to 22 after 7 days (from 17 to 1% of total CHO_x_S_4_ annotations). The CHO_x_S_5_-subspace disappears completely after 3 days. Compounds annotated with 1–2 sulfur show the opposite trend. The percentage of CHO_x_S_1_ increases from 27% to 44.5% over the time of the experiment. We observe an inverted picture for the relative abundance of CHO compounds. CHO compounds with 2–3 oxygen represent 27–34% of all CHO annotations after 2 h of incubation. This percentage is reduced to 13–15% after 7 days of incubation.

**Fig. 6:**
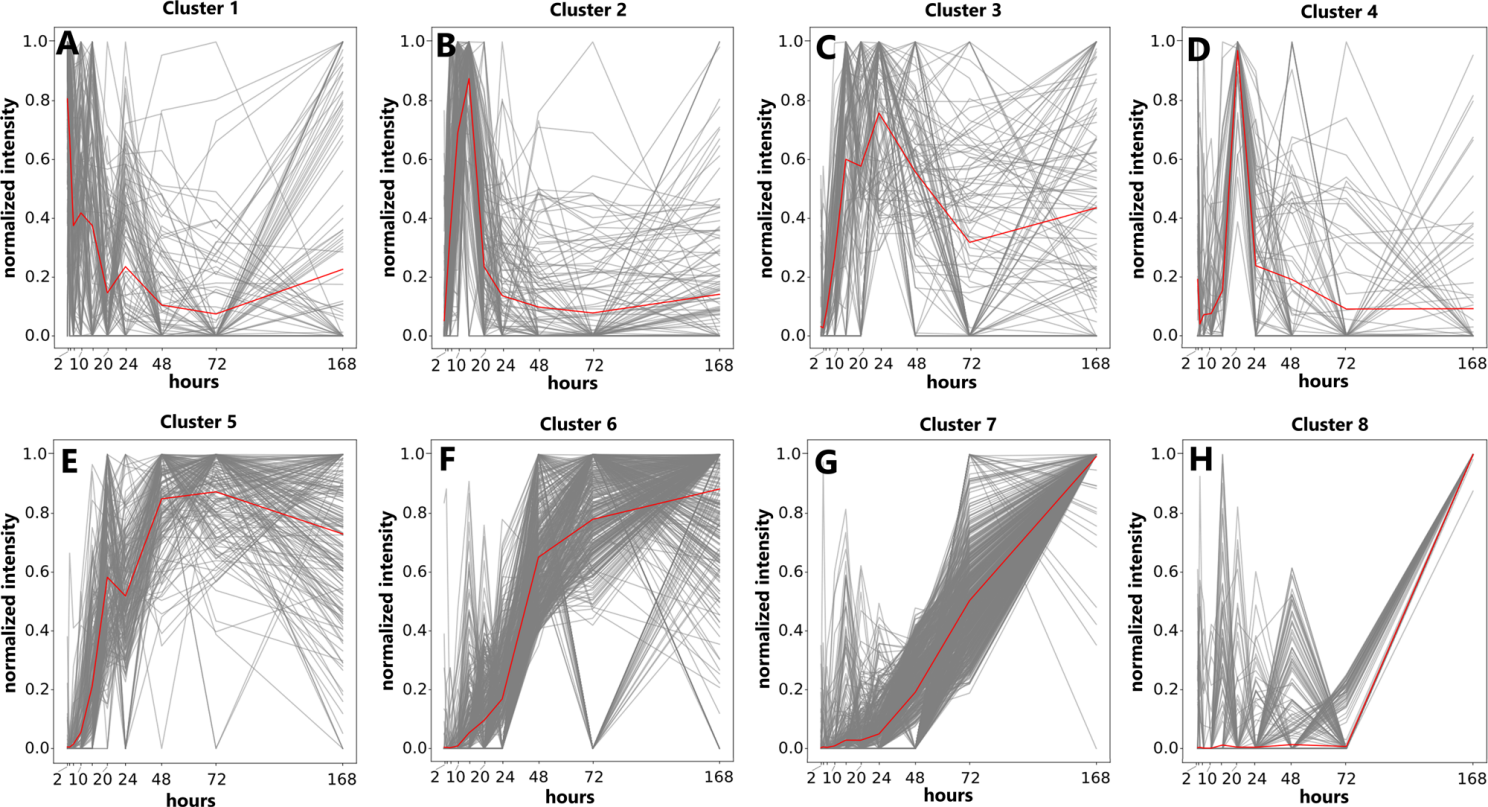
SOM analysis. SOM clusters showing intensity traces for all annotated masses (gray) and an average path taken (red), with different intensity maxima for different clusters. **A**–**G** are ordered by peak time. **A** Cluster 1 peaks after 2 h and decreases over time. **B** Cluster 2 peaks after 15 h. **C** Cluster 3 peaks between 15 and 24 h. **D** Cluster 4 peaks after 20 h. **E** Cluster 5 peaks after 72 h. **F**–**H** Cluster 6–8 shows maximum intensity after 168 h with different slopes.

We have further used self-organizing maps (SOM) classification theory^30^ to characterize time-dependent molecular profiles in greater detail. This technique allows the clustering of temporal mass abundance profiles that follow the same evolutional trend over time. Masses following a similar intensity change over time are clustered together. The approach was already successfully used in other untargeted FT-ICR-MS analyses to categorize the temporal evolution of detected mass signals^31^ in food samples. Our results show 8 different main clusters (Fig. 6). These clusters show groups of masses reaching their maximum intensity at different time points. We can confirm two trends that were suggested in the temporal network.: Clusters with maximum intensity at earlier time points show elemental compositions with lower masses on average than elemental compositions in clusters with late maxima. Compounds with higher amounts of sulfur reach their intensity maximum earlier than compounds with less sulfur. This fact shows that the continuous addition of sulfur to the unsaturated olefinic molecules is unlikely.

**Fig. 7:**
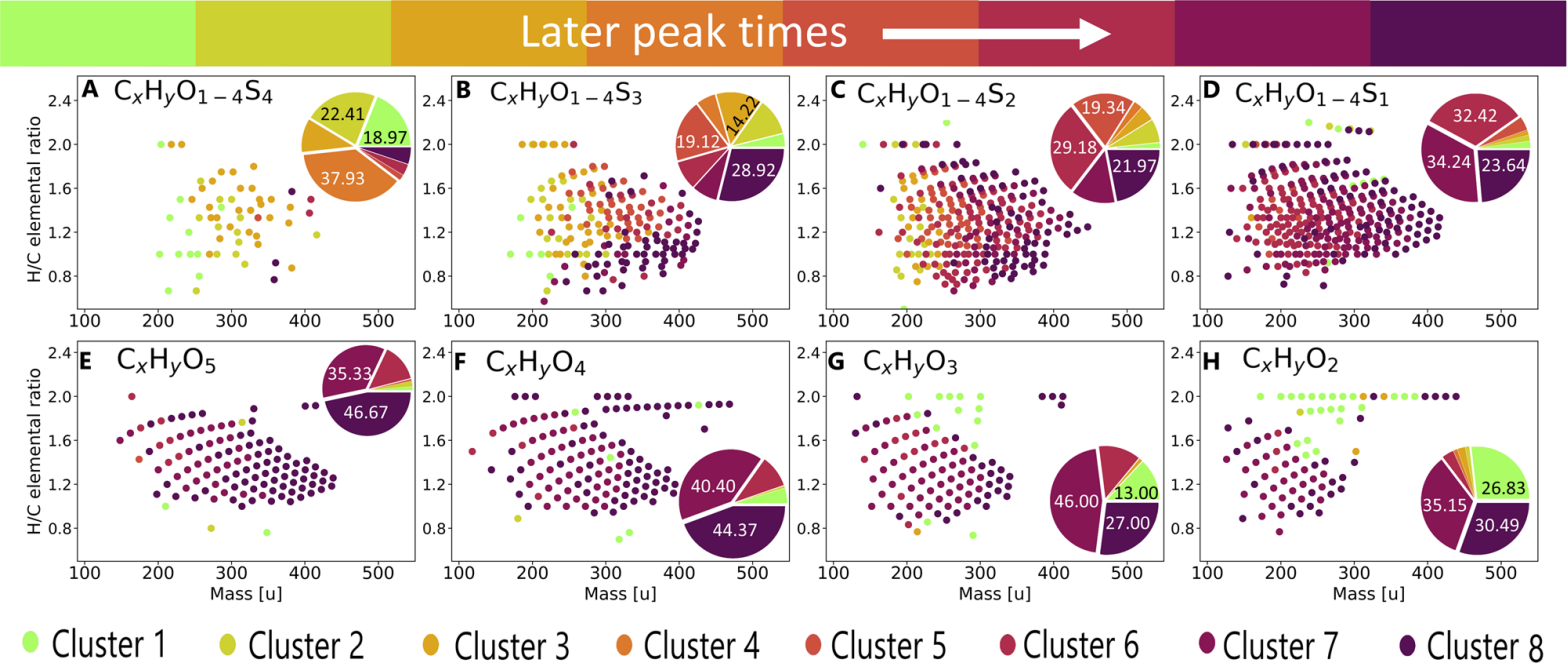
SOM-clustering for different chemical spaces. CHO_1-4_S (panels **A**–**D**) and CHO (pannels **E**–**H**) subspaces colored by their maximum peak intensity through SOM-clustering, showing predominantly earlier peak times for compounds with high sulfur numbers. Green color indicates early peak times and evolves to violet for later emerging compounds. Pie charts on the right side of the plots show the percentage of annotations for the different peak times/clusters.

The profiles of the different groups give insight into the changing reactions over time, as compounds do not all linearly accumulate in the system but have different peak times. Some compounds are mostly depleted after 7 days (Fig. 6A, B, D). The behavior of sulfur over time is particularly interesting (Fig. 7). Sulfur-containing compounds with 3 or 4 sulfur atoms appear earlier and degrade quickly (Fig. 7A, B). The peak time appears later for annotations with lower numbers of sulfur (Fig. 7C, D). This effect differs for CHO compounds, where the compounds have a peak time of 7 days (or later) and mostly show steady signal increases over time.

Based on our results, the addition of acetylene or other mass-increasing reactions compensates for the loss of mass due to lower amounts of sulfur. However, an exchange of sulfur through other mass-increasing building blocks can only be hypothesized. 84% of all annotated thio acids follow the trend of cluster 6 (Fig. 6H). The observed behavior of the higher mass thio acids is different from their low mass counterpart thio acetic acid, which is present after 2 h in high amounts and then reacts away throughout the experiment (supplementary fig. 2), partially by hydrolyzing to acetic acid.

## Discussion

The described system demonstrated its potential in previous publications to produce different prebiotically-relevant substances like aldehydes, fatty acids, and thiophenes^2,17,23^. The formation of pyrrole^19^ and amino acids from aldehydes^17^ via the Strecker reaction was also demonstrated in previously published experiments that included nitrogen. Our study now expands these targeted observations to reveal a dynamic chemical landscape of functional groups derived from basic building blocks, emphasizing sulfur-containing molecules and the deconvolution of functional groups with the help of ^13^C labeling. The size of the investigated molecules also continues to grow progressively over time, mainly via a C2-unit increase, reminiscent of the C2-metabolism including the formation of fatty acids, polyketides, and terpenes from acetyl-CoA units in extant organisms^32^. Some prokaryotes even use acetylene as a main carbon source for their metabolism. The Gram-negative bacterium *Pelobacter acetylenicus* lives in anoxic oceanic sediments and converts acetylene into acetyl-CoA via acetaldehyde to fuel its C2-metabolism^12^. Capitalizing on our previous findings of a primordial conversion of acetylene into fatty acids and related organic molecules, this primitive bacterium could therefore be seen as the link between a purely abiotic acetylene-based C2-metabolism and the C2-metabolism in extant organisms. The formation of acetaldehyde and thio acetic acid S-methyl ester (a simple analog to acetyl-CoA) from acetylene was already shown in this system^16,17^.

Isotope labeling uncovered the system’s hidden diversity in functional groups. We could separate functional groups with the same number of hetero atoms by FT-ICR-MS. Separating functional groups with the same number of hetero atoms is difficult for FT-ICR-MS-based analysis. The problem could be solved by ^13^C isotope labeling of the starting materials. This approach made it possible to trace the origin and chemical nature of hetero atoms based on the literature^1,18,25^. The CHO_3_-subspace shows hydroxy acids and keto acids. Hydroxy acids continue to gain interest in the origin-of-life field. α-hydroxy acids can enhance peptide-bond formation in dry-down reactions^33^, even though our analysis does not allow for the exact identification of the position of the functional group. The same can be said of keto acids, which play a central role in an ancestral analog of the Krebs cycle^34,35^. Dicarboxylic acids gained recent attention due to their ability to form co-polymers with glycol nucleic acids with a hypothesized potential to perform genetic or catalytic functions^36^. The diverse compound classes from multiple species have already proven to be significant in the origin of life.

After the primary introduction of varying amounts of sulfur to the initial reactants, we observed a percentual decrease of sulfur atoms per molecule over time instead of a continuous increase. This decrease in sulfur is true for all sulfur-containing groups that could be resolved and categorized in this study. The behavior of sulfur differs from that of the other elements in the system. The disappearance of sulfur from the formed compounds is an interesting observation. Sulfur represents an important element for origin-of-life reactions. In extant organisms, sulfur is less abundant than other relevant elements like carbon, nitrogen, and phosphate. Nonetheless, the remaining sulfur plays a crucial role in extant organisms. Indeed, thioesters like acetyl-CoA belong to the metabolically essential sulfur molecules. It is challenging to provide conclusive evidence for the precise process that leads to a sulfur reduction in the resulting compounds. Still, it can only be hypothesized with this untargeted approach.

We conducted and analyzed a 7-day experiment to study the evolution of a system with progressively decreasing sulfur levels. This system can be considered a fast motion of early evolution on Earth.

The analysis of the fraction of isomers revealed an unexpected distribution. Instead of a steady increase in isomer diversity, some chemical subspaces, mainly the sulfur-containing ones, showed very high variance in mixed annotations. This result shows an unknown amount of directed synthesis in the system, as some subspaces keep a certain amount of functional purity and do not uncontrollably diversify over time.

It is also relevant to mention the presence of thio acids in a large diversity. The present investigation reveals the full extent of the possible molecular diversity of pure thio acids and describes different formation pathways. One pathway requires acetylene and carbon monoxide; the other relies on acetylene alone. Independent of the pathway, the increase in mass and many saturation levels remain comparable. The temporal analysis of the mixture also revealed that larger thio acids reach their maximum intensity much later than thio acetic acid, most of them belonging to the SOM cluster reaching a maximum after 3 days. Thio acetic acid, on the other hand, decays quickly and becomes undetectable via NMR after 8 h.

Acetylene is the main driver of the increased mass of the detected molecules. Labeled atoms stemming from carbon monoxide remain in the single digits independently of the size of the molecule. Compounds detected after 7 days show between 15 and 27 carbon atoms within the mass range of 230–500 m/z but present only up to four carbon labels from ^13^CO.

Acetylene is still a building block often overlooked. Still, our finding turns it into an important tool to increase molecular mass or as a C2-spacer to optimize the spatial arrangement of functional groups. All compound classes exhibit a noticeable increase in two-carbon mass, known as C2-chemistry. This phenomenon closely resembles the C2-metabolism of contemporary organisms, which is mediated by acetyl-CoA.

## Conclusion

This work revealed a new dimension of complexity in a hydrothermal system based on the “metal-sulfur” world theory. Utilizing isotope labeling to categorize the functional groups enhanced the understanding of the reactions in the system. Unexpected behavior was observed for sulfur, as its number was lowered in detected compounds over time, leading to sulfur numbers more akin to the biological molecules in contemporary organisms. We have discovered several ways to produce thio acids, indicating that this group of compounds is easily accessible in the environment we investigated. Furthermore, the role of acetylene as the main building block for synthesizing higher-mass compounds was shown by differentiating carbon originating from carbon monoxide and acetylene. We revealed a reoccurring pattern of C2-addition to all formed compounds through acetylene, a nutrient used by *Pelobacter acetylenicus* to fuel its fatty acid synthesis via a similar C2-metabolism. These results show new paths for further investigations that require the described functional groups or deliver a framework to tackle the analysis of even more complex systems containing nitrogen or phosphor.

## Methods

### Reaction bottle setup

In a typical run, a 125 ml glass serum bottle was charged with 1.0 mmol NiSO_4_ • 6 H_2_O (99%, Aldrich) and sealed with a silicon stopper. The bottle underwent three cycles of evacuation and argon filling, ultimately reaching a deaerated state. Subsequently, the bottle was filled with argon-saturated water (calculated for the end volume of 5 ml), with 1.0 mL argon-saturated 1 M Na_2_S (solid Na_2_S: 99.99%, Aldrich) solution, with 1.0 mL 1 M NaOH solution and finally with 60 ml unlabeled CO and 60 ml unlabeled acetylene (acetone-free), using gastight syringes for the injections. Reactions were carried out at 105 °C. Following a reaction time of up to 7 days, the reaction mixture was cooled down. To conduct labeling experiments, ^13^CO was utilized, while in a control run with the same composition, acetylene and CO were substituted with argon.

### FT-ICR mass spectrometry

Samples were taken from the serum bottle with a syringe and centrifuged for 5 min at 15000 *rpm*. 100 µl of the supernatant were diluted in 900 µl methanol and centrifuged again to remove the precipitated salt. 70 µl of the centrifuged sample were diluted again in 930 µl methanol. For the timepoints 2 h, 1 day, 2 days, 3 days, 7 days and 7 days (13 C labeled) three different bottles were analyzed as biological replicates. Time points 3 h to 20 h were measured with two biological replicates. Every biological replicate was measured as three technical replicates. Only signals appearing in >66% of a triplicate were kept for annotation. Only annotated signals with a H/C ratio between 0.5 and 2.5 were kept. O/C ratios had to be below 1.5 for all annotations.

Analysis was performed on a high-field Fourier Transform Ion Cyclotron Resonance mass spectrometer from Bruker Daltonics—Solarix with a 12 T magnet from Magnex. The mass spectra were acquired with a 4-megaword (MW) time domain. The system was calibrated with L-Arginine clusters in negative ionization mode (5 mg L^−1^ L-arginine solved in methanol). For each sample, 200 scans were accumulated in negative ion mode in the mass range of 122–1000 amu. Ions were accumulated for 300 ms. The pressure in the hexapole was 3 × 10^−6^ mbar, and the pressure in the ICR vacuum chamber was 6 × 10^−6^ mbar. An Apollo ii (Bruker Daltonics) ESI source was used. The supernatant was injected via a microliter pump system (flow rate: 120 µl h^−1^).

Data were recalibrated post data collection via a calibration list based on fatty acids with different chain lengths. Peaks were picked automatically in Data Analysis (Bruker) with a s/n threshold of 4. Mass lists were exported, filtered with two in-house filters removing wiggles artifacts and natural 34 S isotopes.

Formula assignment was done through a mass difference network approach^37^. The transformation list can be found in the excel sheet supplementary data.

Annotation of the labeling degree was done by comparing the 7-day setup with a CO-^13^C- or acetylene ^13^C labeled 7-day setup. In CO-labeled setups, compounds were categorized as labeled if the corresponding signal in the labeled sample showed a signal that surpassed the expected signal intensity of the natural ^13^C (1% times the number of carbon atoms in an elemental composition) by 100%. ^13^C acetylene signals were checked manually and had to fulfill the same requirements.

Molecular networks were generated and analyzed via the method mol2net (https://zenodo.org/record/7025094; Ruf & Danger 2022).

### SOM-clustering

The data were preprocessed by the sklearn MinMaxScaler function. The SOM model was implemented on a 2 × 4 grid with a learning rate of 0.1. A Gaussian neighborhood function on top of a rectangular topology was used. Euclidean activation distances were used for model calculations. The compiled model was trained for 50,000 iterations. Aggregated clusters were extracted from the winning map and plotted with respective averaged cluster centers.

### Supplementary information

Supplementary Material

Description of Additional Supplementary Files

Supplementary Data 1

## Data Availability

The authors declare that [the/all other] data supporting the findings of this study are available within the paper [and in Supplementary Data 1]. Further data that support the findings of this study are available from the corresponding author upon reasonable request.

## References

[1] Reppe W (1953). Carbonylierung I. Über die Umsetzung von Acetylen mit Kohlenoxyd und Verbindungen mit reaktionsfähigen Wasserstoffatomen Synthesen α,β-ungesättigter Carbonsäuren und ihrer Derivate. Justus Liebigs Ann. der Chem..

[2] Scheidler C, Sobotta J, Eisenreich W, Wächtershäuser G, Huber C (2016). Unsaturated C3,5,7,9-Monocarboxylic Acids by Aqueous, One-Pot Carbon Fixation: Possible Relevance for the Origin of Life. Sci. Rep..

[3] Ormeland, R. S. & Voytek, M. A. Acetylene as Fast Food: Implications for Development of Life on Anoxic Primordial Earth and in the Outer Solar System. *Astrobiology* **8**, 45–58 (2008). 10.1089/ast.2007.0183 18199006

[4] Abbas O. & Schulze-Makuch D. Acetylene-based pathways for prebiotic evolution on Titan. *Exo-Astrobiology* **518**, 345–348 (2002).

[5] Cable ML, Vu TH, Maynard-Casely HE, Choukroun M, Hodyss R (2018). The Acetylene-Ammonia Co-crystal on Titan. ACS Earth Space Chem..

[6] Melin H, Fletcher LN, Irwin PGJ, Edgington SG (2020). Jupiter in the Ultraviolet: Acetylene and Ethane Abundances in the Stratosphere of Jupiter from Cassini Observations between 0.15 and 0.19 μm. Astronom. J..

[7] Postberg F (2023). Detection of phosphates originating from Enceladus’s ocean. Nature.

[8] Waite JH (2006). Cassini ion and neutral mass spectrometer: Enceladus plume composition and structure. Science.

[9] Rimmer PB, Shorttle O (2019). Origin of Life’s Building Blocks in Carbon- and Nitrogen-Rich Surface Hydrothermal Vents. Life.

[10] Capaccioni B (1993). Light hydrocarbons in gas-emissions from volcanic areas and geothermal fields. Geochem. J..

[11] Sanchez RA, Ferris JP, Orgel LE (1966). Cyanoacetylene in Prebiotic Synthesis. Science.

[12] Schink B (1985). Fermentation of acetylene by an obligate anaerobe,Pelobacter acetylenicus sp. nov. Arch. Microbiol..

[13] Diender M, Stams AJM, Sousa DZ (2015). Pathways and Bioenergetics of Anaerobic Carbon Monoxide Fermentation. Front. Microbiol..

[14] Wächtershäuser G (1988). Before enzymes and templates: theory of surface metabolism. Microbiol. Rev..

[15] Wächtershäuser G (1990). Evolution of the first metabolic cycles. Proc. Natl Acad. Sci..

[16] Huber C, Wächtershäuser G (1997). Activated acetic acid by carbon fixation on (Fe,Ni)S under primordial conditions. Science.

[17] Diederich P (2023). Formation, stabilization and fate of acetaldehyde and higher aldehydes in an autonomously changing prebiotic system emerging from acetylene. Commun. Chem..

[18] Sobotta J (2020). A Possible Primordial Acetyleno/Carboxydotrophic Core Metabolism. Life.

[19] Seitz C., Eisenreich W. & Huber C. The Abiotic Formation of Pyrrole under Volcanic, Hydrothermal Conditions—An Initial Step towards Life’s First Breath? *Life* **11**, 980 (2021). 10.3390/life11090980 PMC8471139 34575129

[20] de Duve C. Chapter 7 Harnessing Energy. In: *Blueprint for a cell: the nature and origin of life*. (N. Patterson in 1991).

[21] Frenkel-Pinter M (2022). Thioesters provide a plausible prebiotic path to proto-peptides. Nat. Commun..

[22] Closs AC, Bechtel M, Trapp O (2022). Dynamic Exchange of Substituents in a Prebiotic Organocatalyst: Initial Steps towards an Evolutionary System. Angew. Chem. Int. Ed..

[23] Geisberger T, Sobotta J, Eisenreich W, Huber C (2021). Formation of Thiophene under Simulated Volcanic Hydrothermal Conditions on Earth—Implications for Early. Life Extraterrest. Planets? Life.

[24] Giavalisco P (2008). High-Resolution Direct Infusion-Based Mass Spectrometry in Combination with Whole 13C Metabolome Isotope Labeling Allows Unambiguous Assignment of Chemical Sum Formulas. Anal. Chem..

[25] Nieuwland JA, Calcott WS, Downing FB, Carter AS (1931). Acetylene polymers and their derivatives. I. The controlled polymerization of acetylene. J. Am. Chem. Soc..

[26] Kutscheroff M (1881). Ueber eine neue Methode direkter Addition von Wasser (Hydratation) an die Kohlenwasserstoffe der Acetylenreihe. Ber. der Dtsch. Chem. Ges..

[27] Jones SO, Reid EE (1938). The Addition of Sulfur, Hydrogen Sulfide and Mercaptans to Unsaturated Hydrocarbons. J. Am. Chem. Soc..

[28] Moritz F (2015). The compositional space of exhaled breath condensate and its link to the human breath volatilome. J. Breath. Res..

[29] Ruf A, Danger G (2022). Network Analysis Reveals Spatial Clustering and Annotation of Complex Chemical Spaces: Application to Astrochemistry. Anal. Chem..

[30] Kohonen T (1982). Self-organized formation of topologically correct feature maps. Biol. Cybern..

[31] Weidner L, Hemmler D, Rychlik M, Schmitt-Kopplin P (2023). Real-Time Monitoring of Miniaturized Thermal Food Processing by Advanced Mass Spectrometric Techniques. Anal. Chem..

[32] Cronan J. E., Thomas J. Chapter 17 Bacterial Fatty Acid Synthesis and its Relationships with Polyketide Synthetic Pathways. In: *Methods in Enzymology*). (Academic Press, 2009). 10.1016/S0076-6879(09)04617-5 PMC4095770 19362649

[33] Frenkel-Pinter M, Sargon AB, Glass JB, Hud NV, Williams LD (2021). Transition metals enhance prebiotic depsipeptide oligomerization reactions involving histidine. RSC Adv..

[34] Stubbs RT, Yadav M, Krishnamurthy R, Springsteen G (2020). A plausible metal-free ancestral analogue of the Krebs cycle composed entirely of α-ketoacids. Nat. Chem..

[35] Pulletikurti S, Yadav M, Springsteen G, Krishnamurthy R (2022). Prebiotic synthesis of α-amino acids and orotate from α-ketoacids potentiates transition to extant metabolic pathways. Nat. Chem..

[36] Yi R (2023). Alternating co-synthesis of glycol nucleic acid (GNA) monomers with dicarboxylic acids via drying. Chem. Commun..

[37] Tziotis D, Hertkorn N, Schmitt-Kopplin P (2011). Kendrick-Analogous Network Visualisation of Ion Cyclotron Resonance Fourier Transform Mass Spectra: Improved Options for the Assignment of Elemental Compositions and the Classification of Organic Molecular Complexity. Eur. J. Mass Spectrom..

