# Supplementary material for: C2-addition patterns emerging from acetylene and nickel sulfide in simulated prebiotic hydrothermal conditions
Source: Commun Chem. 2023 Oct 12;6:220. doi: 10.1038/s42004-023-01021-1 (PMC10570370; doi:10.1038/s42004-023-01021-1)
Supplement: Supplementary file 1 — Supplementary Material [file 42004_2023_1021_MOESM1_ESM.pdf]

Supplementary Information

C2-addition patterns emerging from acetylene and nickel sulfide in simulated prebiotic hydrothermal conditions

Philippe Diederich [a], Alexander Ruf [b,c], Thomas Geisberger [d], Leopold Weidner [a,e] Christian Seitz [d], Wolfgang Eisenreich [d], Claudia Huber [d*], Philippe Schmitt-Kopplin [a, e, f*]

[a] Helmholtz Munich, Research Unit Analytical BioGeoChemistry, Neuherberg, Germany.

[b] Excellence Cluster ORIGINS, Boltzmannstraße 2, 85748 Garching, Germany
[c] LMU Munich, Faculty of Physics, Schellingstraße 4, 80799 Munich, Germany
[d] Technical University of Munich, TUM School of Natural Sciences, Department of Bioscience, Bavarian NMR Center (BNMRZ), Structural Membrane Biochemistry, Lichtenbergstr. 4, 85748 Garching, Germany

[e] Comprehensive Foodomics Platform, Chair of Analytical Food Chemistry, TUM School of Life Sciences, Technical University of Munich, Maximus-von-Imhof-Forum 2, 85354 Freising, Germany

[f] Max Planck Institute for Extraterrestrial Physics, Center for Astrochemical Studies, Gießebachstraße 1, 85748 Garching bei München, Germany.

*Corresponding authors: Research Unit Analytical BioGeoChemistry, Helmholtz Zentrum München–German Research Center for Environmental Health, Neuherberg, Germany and Technical University of Munich, TUM School of Natural Sciences, Department of Bioscience, Bavarian NMR Center (BNMRZ), Structural Membrane Biochemistry, Lichtenbergstr. 4, 85748 Garching, Germany

Emails: schmitt-kopplin@helmholtz-muenchen.de, claudia.huber@tum.de

Correlation between number of oxygen atoms per molecule and the H/C ratio for different compound classes

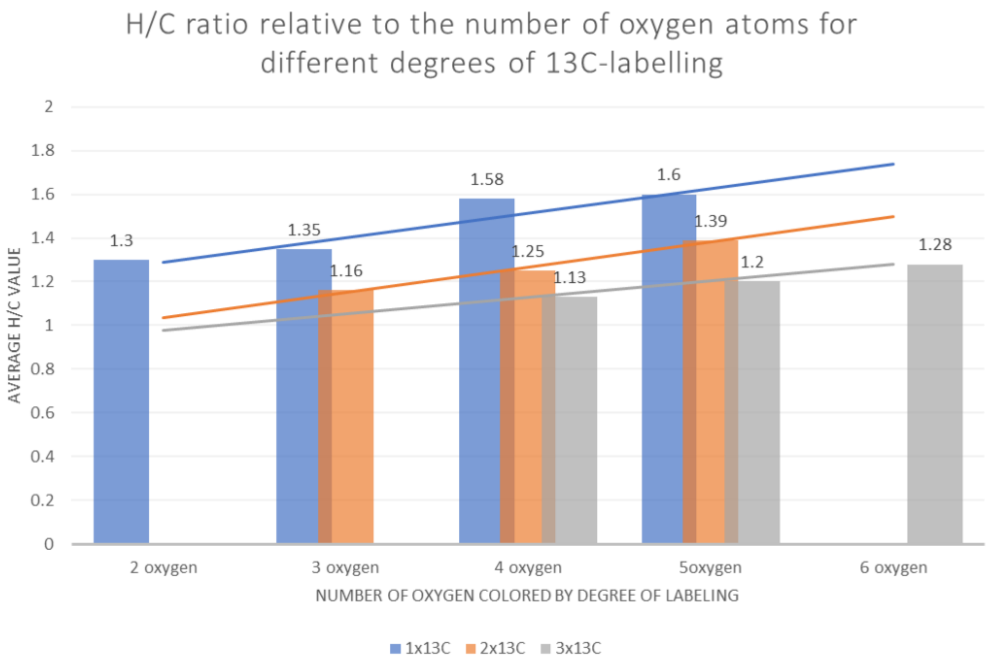

Supplementary Figure 1: Barplot showing the increasing H/C ratio with increasing amounts of oxygen atoms per molecule.

Spiking experiment with thio acetic acid standard

To prove the presence of thio acetic acid at early time points and its disappearance later, we measured the mixture after 2 hours and 7 days. To confirm the presence of thio acetic acid, a standard was spiked to the 7-day sample, and its chemical shift was compared to the signal after 2 hours (Supplementary Figure 1, gray line).

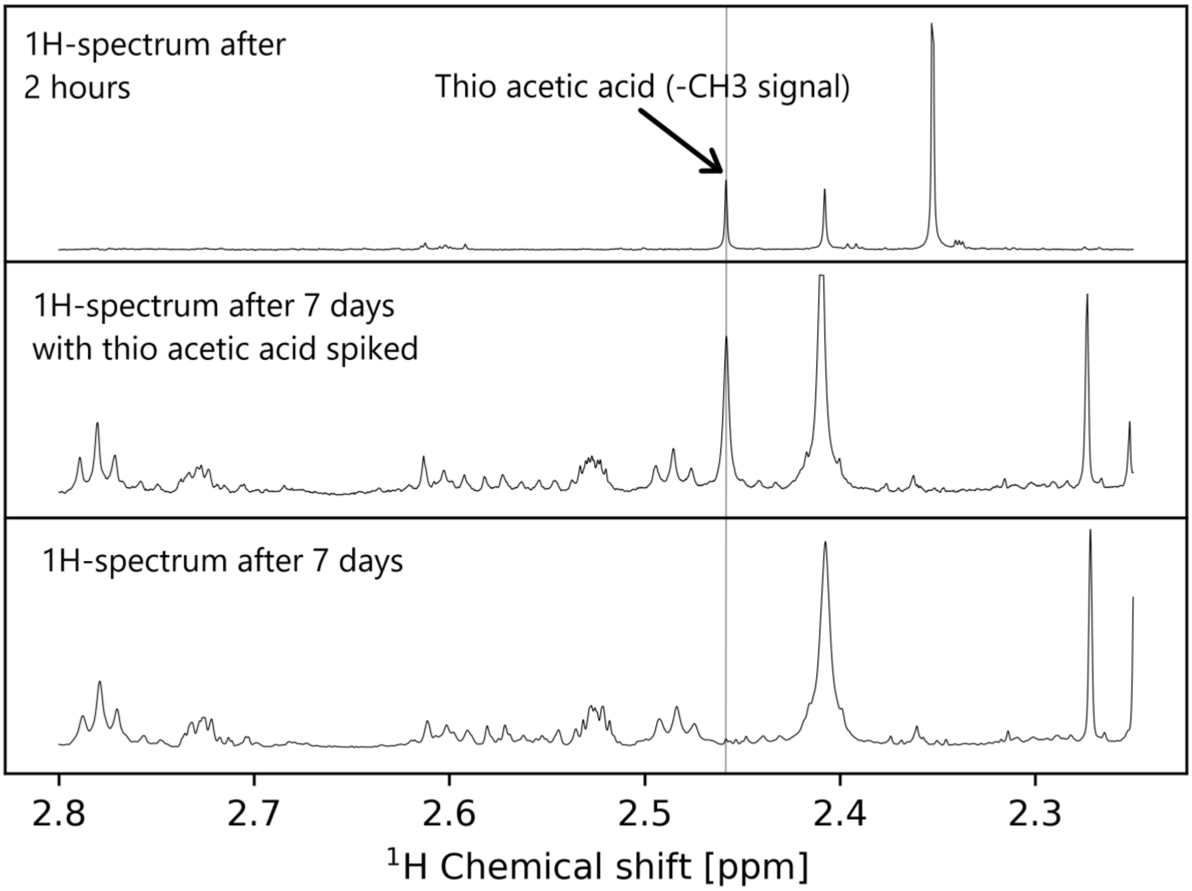

Supplementary Figure 2: ^{1}H-spectrum of the investigated setup after 2 hours and 7 days. Thio acetic acid was spiked into the 7-day experiment to confirm this compound. The gray line marks the chemical shift of thio acetic acid.

Identification of 3,3´-thiobispropanoic acid

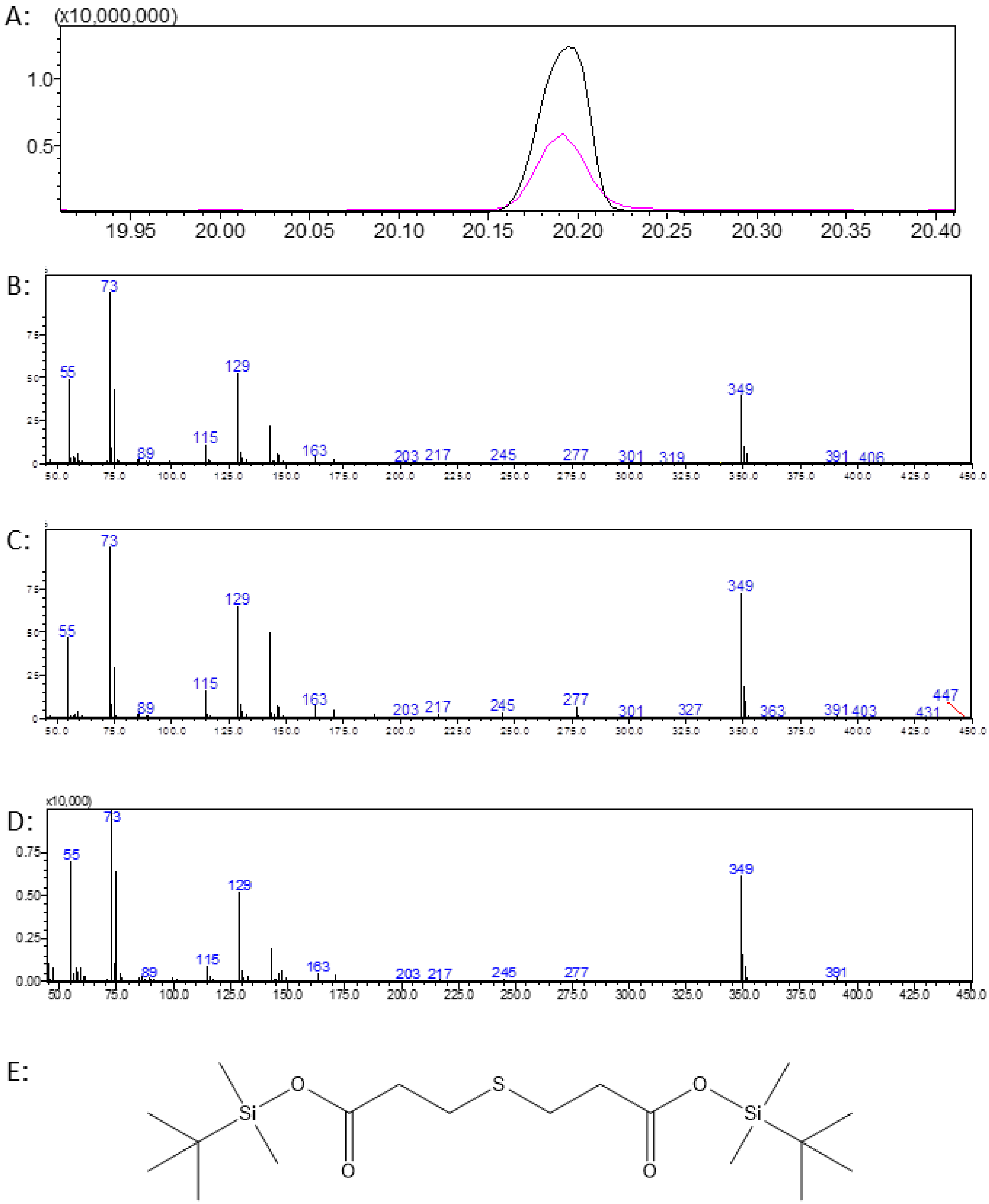

Supplementary Figure 3: GC-MS chromatogram and mass spectra from TBDMS derivatized 3,3´-thiobispropanoic acid. Sample preparation and GC-MS conditions are described in [15]

A: Chromatogram showing mass traces from a commercially available standard (black) and 3,3´-thiobispropanoic acid from the reaction supernatant (pink).

B: Mass spectrum from the commercially available 3,3´-thiobispropanoic acid

C: Mass spectrum from the reaction supernatant

D: Mass spectrum taken from library NIST14.

E: Structure of TBDMS derivatized 3,3´-thiobispropanoic acid (characteristic fragments: M-15 (methyl group): m/z 391; M-57 (tert-butyl group): m/z 349

Higher sulfur compounds showing homologous series of sodium adducts

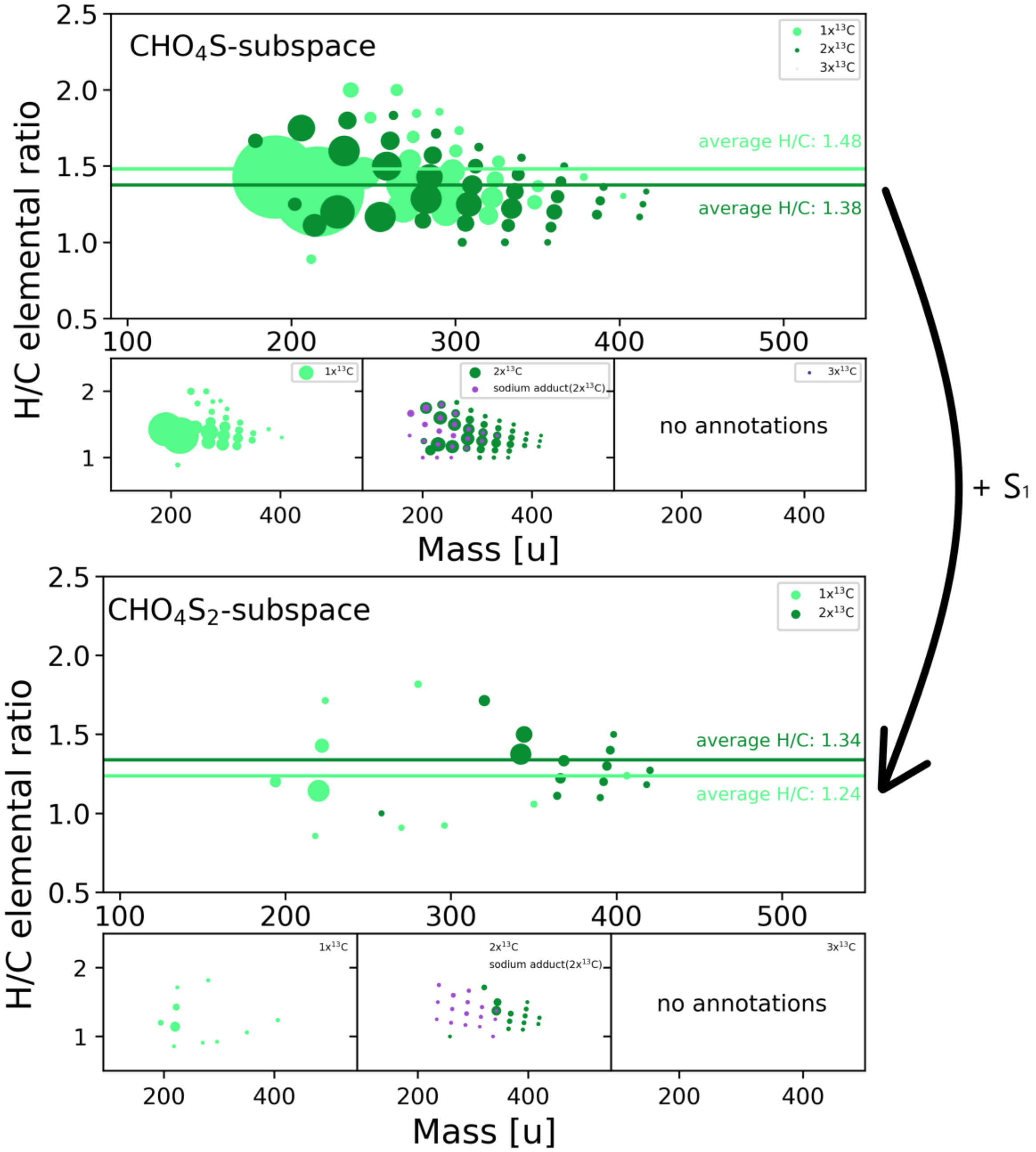

Supplementary Figure 4: Comparison of CHO_4S-subspace and CHO_4S_2-subspace. The two subspaces show interesting similarities to the CHO_4-subspace, mainly consisting of dicarboxylic acids. Comparing the two sulfur subspaces shows the nearly identical level of saturation and labeling between the fractions of sodium adducts (violet). This suggests that they are structurally very similar and are potentially only differentiated by a longer sulfur chain.

Proposed reaction schemes for the categorized compound classes

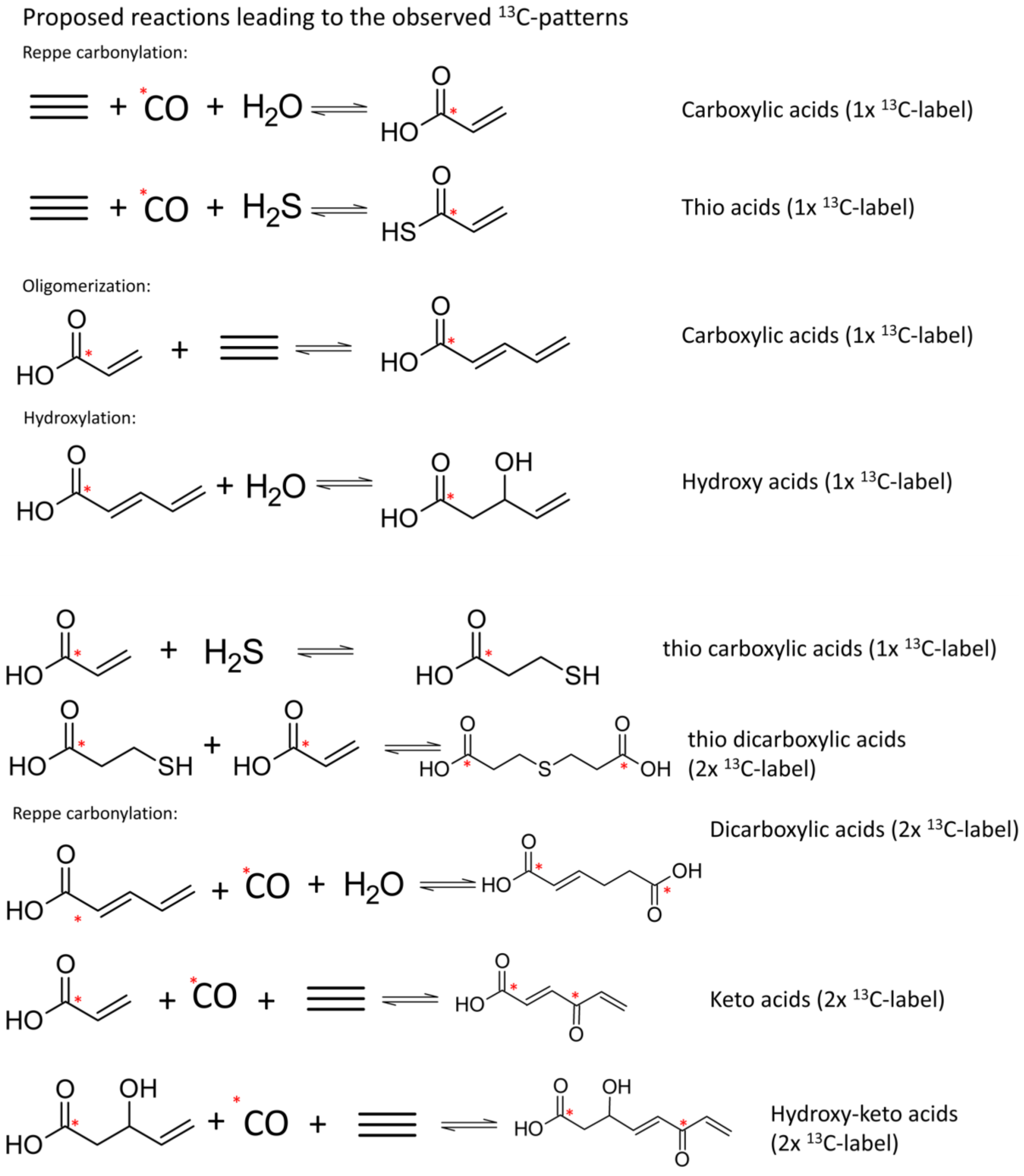

Supplementary Figure 5: Reactions with carbon monoxide leading to different degrees of ^{13}C-labeling

Spectral manifestation of 13C-labeling. Unlabeled setup compared to labeled carbon monoxide spectrum

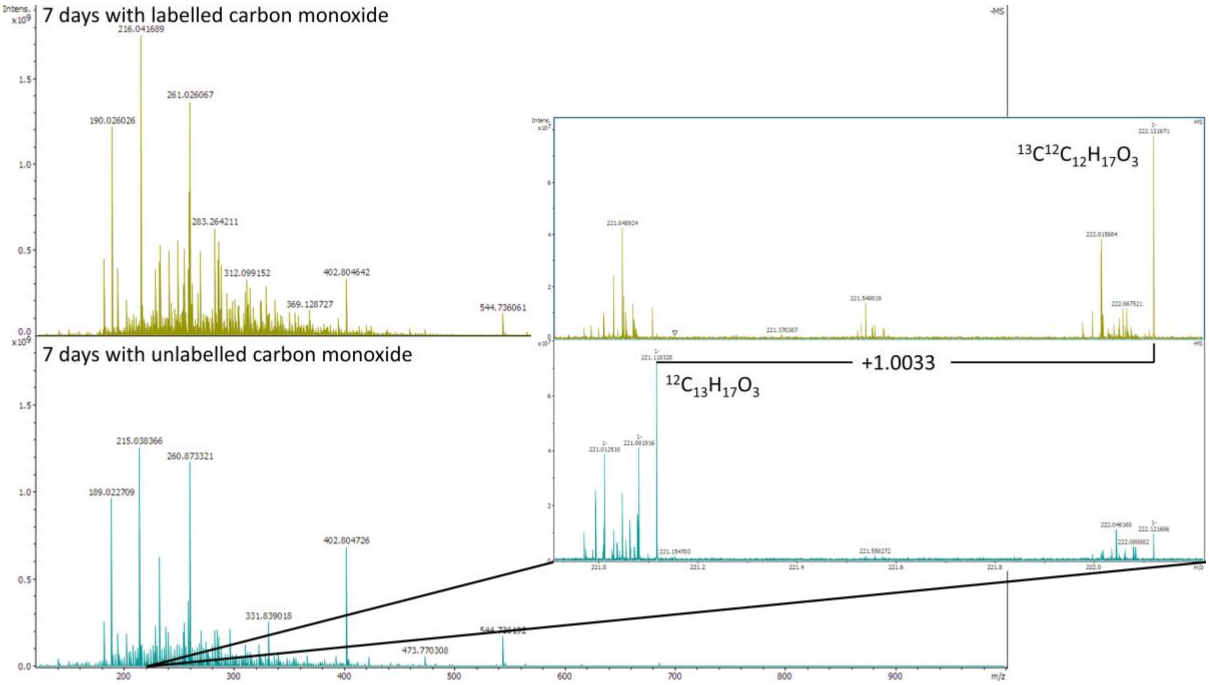

Supplementary Figure 6: Figure showing the spectral appearance of an unlabeled setup versus a lebaled setup. The magnification shows how the mass of a labeled signal shifts by exactly 1.0033 amu.
