# Supplementary material for: C2-addition patterns emerging from acetylene and nickel sulfide in simulated prebiotic hydrothermal conditions
Source: Commun Chem. 2023 Oct 12;6:220. doi: 10.1038/s42004-023-01021-1 (PMC10570370; doi:10.1038/s42004-023-01021-1)
Supplement: Supplementary file 2 — Description of Additional Supplementary Files [file 42004_2023_1021_MOESM2_ESM.pdf]

Description of Additional Supplementary Files

File name: Supplementary Data 1

Description: Supplementary Data 1 contains three sheets named "SOM_and_13C", "All_replicates" and "Transformation_list".

- "SOM_and_13C" contains the average intensity detected for each assigned elemental composition, its assigned SOM cluster determined by the SOM algorithm and its determined degree of 13C-labelling.
- "All_replicates" contains every sample measured with the corresponding intensities for every assigned elemental composition.
- "Transformation_list" contains the mass differences used to do the assignement of the elemental composition via a network approach.
